# Chlorido{*N*-[(diethyl­amino)­dimethyl­sil­yl]anilido-κ*N*}(*N*,*N*,*N*′,*N*′-tetra­methyl­ethane-1,2-diamine-κ^2^ *N*,*N*′)iron(II)

**DOI:** 10.1107/S1600536812044741

**Published:** 2012-11-03

**Authors:** Juan Chen

**Affiliations:** a Department of Chemistry, Taiyuan Teachers College, Taiyuan 030031, People’s Republic of China

## Abstract

In the title iron(II) complex, [Fe(C_12_H_21_N_2_Si)Cl(C_6_H_16_N_2_)], the Fe^II^ cation is coordinated by two N atoms from the tetramethylethane-1,2-diamine ligand [Fe—N = 2.191 (5) and 2.215 (4) Å], one N atom from the *N*-[(diethyl­amino)­dimethyl­sil­yl]anilide ligand [Fe—N = 1.943 (4) Å] and a chloride ligand [Fe—Cl = 2.2798 (16) Å] in a distorted tetra­hedral geometry. The N—Si—N angle is 113.9 (3)°. The crystal packing exhibits no short inter­molecular contacts.

## Related literature

For Fe^II^ complexes with *N*-donor ligand and utility in fixation of dinitro­gen, see: Smith *et al.* (2001 ▶); Rodriguez *et al.* (2011 ▶). For reviews of related metal amides, see: Holm *et al.* (1996 ▶); Kempe (2000 ▶). For catalytic applications of the related *N*-silylated anilido group 4 metal compounds towards olefin polymerization, see: Gibson *et al.* (1998 ▶); Hill & Hitchcock (2002 ▶); Yuan *et al.* (2010 ▶). For related organometallic compounds with analogous anilido ligands, see: Schumann *et al.* (2000 ▶); Chen (2008 ▶, 2009 ▶).
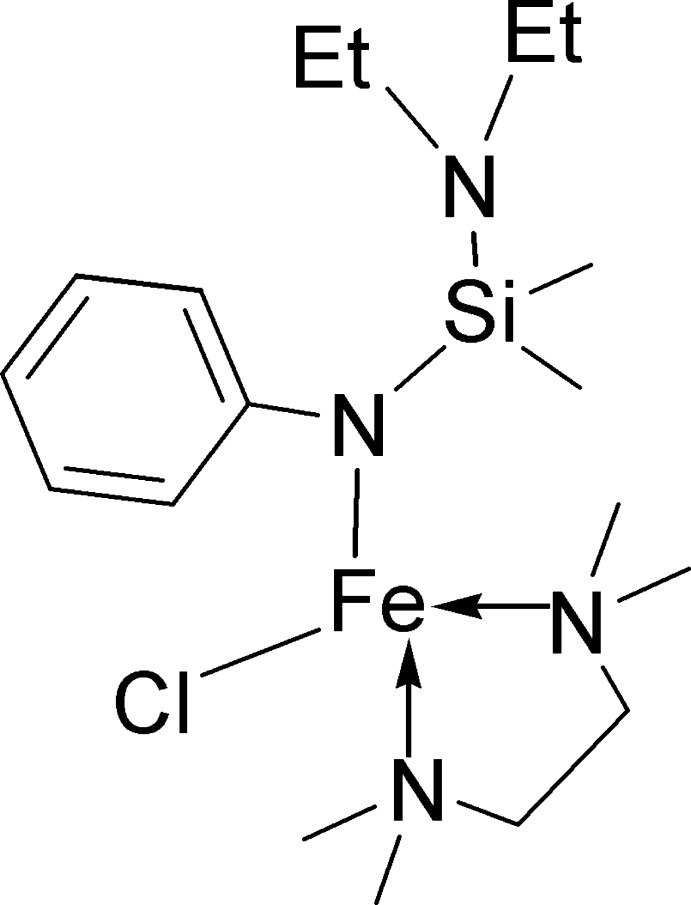


## Experimental

### 

#### Crystal data

[Fe(C_12_H_21_N_2_Si)Cl(C_6_H_16_N_2_)]
*M* *_r_* = 428.91
Monoclinic, 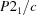

*a* = 16.2317 (10) Å
*b* = 10.7821 (6) Å
*c* = 14.2098 (8) Å
β = 105.157 (1)°
*V* = 2400.4 (2) Å^3^
*Z* = 4
Mo *K*α radiation
μ = 0.80 mm^−1^
*T* = 293 K
0.25 × 0.20 × 0.15 mm

#### Data collection

Bruker SMART area-detector diffractometer
Absorption correction: multi-scan (*SADABS*; Sheldrick, 1996 ▶) *T*_min_ = 0.826, *T*_max_ = 0.890
12665 measured reflections
4222 independent reflections
2584 reflections with *I* > 2σ(*I*)
*R*_int_ = 0.053

#### Refinement

*R*[*F* ^2^ > 2σ(*F* ^2^)] = 0.060
*wR*(*F* ^2^) = 0.190
*S* = 1.02
4222 reflections
226 parameters
14 restraints
H-atom parameters constrained
Δρ_max_ = 0.71 e Å^−3^
Δρ_min_ = −0.65 e Å^−3^

### 

Data collection: *SMART* (Bruker, 2000 ▶); cell refinement: *SAINT* (Bruker, 2000 ▶); data reduction: *SAINT*; program(s) used to solve structure: *SHELXS97* (Sheldrick, 2008 ▶); program(s) used to refine structure: *SHELXL97* (Sheldrick, 2008 ▶); molecular graphics: *SHELXTL/PC* (Sheldrick, 2008 ▶); software used to prepare material for publication: *SHELXL97*.

## Supplementary Material

Crystal structure: contains datablock(s) I, global. DOI: 10.1107/S1600536812044741/cv5351sup1.cif

Structure factors: contains datablock(s) I. DOI: 10.1107/S1600536812044741/cv5351Isup2.hkl

Additional supplementary materials:  crystallographic information; 3D view; checkCIF report

## Figures and Tables

**Fig. 1.**
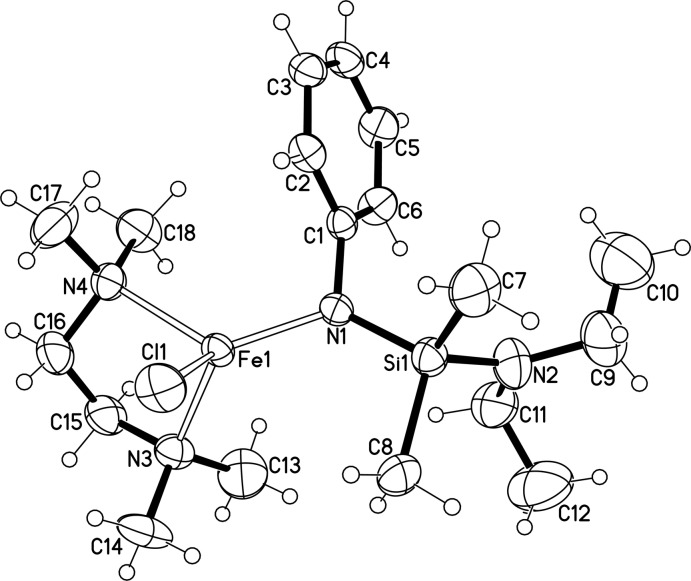
The molecular structure of (I) showing the atom-numbering scheme. Displacement ellipsoids are drawn at the 30% probability level.

## supplementary crystallographic information

### Comment

Metal amides have valuable applications in various industrial and biological processes (Holm *et al.*, 1996; Kempe, 2000). Group 4 metals amides supported with the *N*-silylated anilido ligands are active catalysts for olefin polymerization (Gibson *et al.*, 1998; Hill & Hitchcock, 2002). Moreover, a class of monoionic *N*-silylated anilido ligands bearing a pendant amino group were paid much attentions. It was presumed that the empty *d*-orbitals on silicon would interact with the lone-pair electrons on the *p*-orbital of nitrogen center through *d—pπ* interaction throughout the N—Si—N motif. Analogous compounds with different metals including Zn (Schumann *et al.*, 2000) and Zr (Chen, 2009) have been synthesized. A group of zirconium amides with the similar ligand were reported showing good performance in ethylene polymerization (Yuan *et al.*, 2010). On the other hand, some iron(II) complexes with the *N*-donor ligands were active in fixation of dinitrogen (Smith *et al.*, 2001; Rodriguez *et al.*, 2011). Here, the synthesis and crystal structure of a new iron(II) anilido-complex will be described.

The title compound, (I), was prepared by a one-pot reaction of *n*-LiBu, *N*-[(diethylamino)dimethylsilyl]aniline, 1,2-bis(dimethylamino)ethane (*tmeda*) and FeCl_2_. The suitable for X-ray investigation single-crystal of (I) was obtained by recrystallization in toluene. In (I) , the metal Fe center is coordinated by a chlorido ligand, a chelating *tmeda* molecule and the anilido-ligand. The neutral donor molecule coordinates metal center in *N*,*N'*-chelating mode. Though the anilido-ligand has a pendant amino group, exhibting an N—Si—N chelating moiety, it connects Fe(II) only with a σ-bond, Fe—N_anilide_ being 1.943 (4) Å. It suggests the less affinity between the pendant amino-group and the metal center in comparing with *tmeda*. The N1—Si1—N2 angle is 113.9 (3)°. The four-coordinate Fe atom demonstrates a slightly distorted tetrahedral geometry. In an iron(III) complex with the similar ligand, the N—Si—N unit bit the Fe^III^ metal center and the angle was 95.49 (9)° (Chen, 2008).

### Experimental

A solution of *n*-LiBu (1.6 *M*, 2.1 ml, 3.3 mmol) in hexane was slowly added into a mixture of *N*-[(diethylamino)dimethylsilyl]aniline (0.73 g, 3.3 mmol) and *tmeda* (0.38 g, 3.3 mmol) in *Et*_2_O (20 ml) at 273 K by syringe. The mixture was stirred at room temperature for two hours and then added to a stirring suspension of FeCl_2_ (0.42 g, 3.3 mmol) in *Et*_2_O (20 ml) at 273 K. The resulting mixture was stirred at room temperature for 8 h. Then all the volatiles were removed under vacuum. The residue was extracted with toluene (25 ml). The filtrate was concentrated to 2 ml to yield the title compound as colorless crystals (yield 1.07 g, 76%; m.p. 357–358 K). MS (EI, 70 eV): *m/z* 429 [*M*]^+^. Anal. Calc. for C_18_H_37_ClFeN_4_Si: C, 50.40; H, 8.70; N, 13.06%. Found: C, 50.08; H, 8.61; N, 12.98%.

### Refinement

The methyl H atoms were constrained to an ideal geometry, with C—H distances of 0.96 Å and *U*_iso_(H) = 1.5*U*_eq_(C), but each group was allowed to rotate freely about its C–C, C–N and C–Si bonds. The methylene H atoms were constrained with C—H distances of 0.97 Å and *U*_iso_(H) = 1.2*U*_eq_(C). The phenyl H atoms were placed in geometrically idealized positions and constrained to ride on their parent atoms, with C—H distances in the range 0.93 Å and *U*_iso_(H) = 1.2*U*_eq_(C).

### Crystal data

**Table:**

[Fe(C_12_H_21_N_2_Si)Cl(C_6_H_16_N_2_)]	*F*(000) = 920
*M**_r_* = 428.91	*D*_x_ = 1.187 Mg m^−^^3^
Monoclinic, *P*2_1_/*c*	Mo *K*α radiation, λ = 0.71073 Å
Hall symbol: -P 2ybc	Cell parameters from 3162 reflections
*a* = 16.2317 (10) Å	θ = 2.2–25.3°
*b* = 10.7821 (6) Å	µ = 0.80 mm^−^^1^
*c* = 14.2098 (8) Å	*T* = 293 K
β = 105.157 (1)°	Block, colourless
*V* = 2400.4 (2) Å^3^	0.25 × 0.20 × 0.15 mm
*Z* = 4	

### Data collection

**Table:**

Bruker SMART area-detector diffractometer	4222 independent reflections
Radiation source: fine-focus sealed tube	2584 reflections with *I* > 2σ(*I*)
Graphite monochromator	*R*_int_ = 0.053
φ and ω scan	θ_max_ = 25.0°, θ_min_ = 2.3°
Absorption correction: multi-scan (*SADABS*; Sheldrick, 1996)	*h* = −19→14
*T*_min_ = 0.826, *T*_max_ = 0.890	*k* = −11→12
12665 measured reflections	*l* = −15→16

### Refinement

**Table:**

Refinement on *F*^2^	Primary atom site location: structure-invariant direct methods
Least-squares matrix: full	Secondary atom site location: difference Fourier map
*R*[*F*^2^ > 2σ(*F*^2^)] = 0.060	Hydrogen site location: inferred from neighbouring sites
*wR*(*F*^2^) = 0.190	H-atom parameters constrained
*S* = 1.02	*w* = 1/[σ^2^(*F*_o_^2^) + (0.1016*P*)^2^ + 1.3944*P*] where *P* = (*F*_o_^2^ + 2*F*_c_^2^)/3
4222 reflections	(Δ/σ)_max_ = 0.001
226 parameters	Δρ_max_ = 0.71 e Å^−^^3^
14 restraints	Δρ_min_ = −0.65 e Å^−^^3^

### Special details

**Table:**

Geometry. All e.s.d.'s (except the e.s.d. in the dihedral angle between two l.s. planes) are estimated using the full covariance matrix. The cell e.s.d.'s are taken into account individually in the estimation of e.s.d.'s in distances, angles and torsion angles; correlations between e.s.d.'s in cell parameters are only used when they are defined by crystal symmetry. An approximate (isotropic) treatment of cell e.s.d.'s is used for estimating e.s.d.'s involving l.s. planes.
Refinement. Refinement of *F*^2^ against ALL reflections. The weighted *R*-factor *wR* and goodness of fit *S* are based on *F*^2^, conventional *R*-factors *R* are based on *F*, with *F* set to zero for negative *F*^2^. The threshold expression of *F*^2^ > σ(*F*^2^) is used only for calculating *R*-factors(gt) *etc*. and is not relevant to the choice of reflections for refinement. *R*-factors based on *F*^2^ are statistically about twice as large as those based on *F*, and *R*- factors based on ALL data will be even larger.

### Fractional atomic coordinates and isotropic or equivalent isotropic displacement parameters (Å^2^)

**Table:**

	*x*	*y*	*z*	*U*_iso_*/*U*_eq_	
Fe1	0.82680 (5)	0.53246 (6)	0.32605 (5)	0.0438 (3)	
Si1	0.67995 (11)	0.45040 (15)	0.42532 (12)	0.0577 (5)	
Cl1	0.89234 (12)	0.69105 (14)	0.42333 (13)	0.0771 (5)	
N1	0.7543 (3)	0.4097 (4)	0.3648 (3)	0.0482 (11)	
N2	0.5867 (4)	0.3658 (6)	0.3912 (5)	0.0988 (17)	
N3	0.7721 (3)	0.6122 (4)	0.1794 (3)	0.0625 (13)	
N4	0.9258 (3)	0.4720 (4)	0.2568 (3)	0.0591 (12)	
C1	0.7874 (3)	0.2877 (4)	0.3770 (3)	0.0434 (12)	
C2	0.8678 (4)	0.2640 (5)	0.4374 (4)	0.0542 (14)	
H2A	0.8987	0.3286	0.4734	0.065*	
C3	0.9035 (4)	0.1458 (5)	0.4455 (4)	0.0598 (15)	
H3A	0.9585	0.1331	0.4847	0.072*	
C4	0.8583 (5)	0.0484 (5)	0.3961 (5)	0.0659 (17)	
H4A	0.8816	−0.0309	0.4024	0.079*	
C5	0.7778 (5)	0.0696 (5)	0.3371 (5)	0.0732 (19)	
H5A	0.7464	0.0038	0.3032	0.088*	
C6	0.7430 (4)	0.1871 (5)	0.3275 (4)	0.0622 (16)	
H6A	0.6885	0.1992	0.2869	0.075*	
C7	0.7246 (5)	0.4311 (8)	0.5602 (5)	0.095 (2)	
H7A	0.7376	0.3452	0.5749	0.142*	
H7B	0.6833	0.4585	0.5933	0.142*	
H7C	0.7757	0.4795	0.5816	0.142*	
C8	0.6537 (5)	0.6178 (6)	0.3978 (6)	0.084 (2)	
H8A	0.6307	0.6283	0.3288	0.126*	
H8B	0.7046	0.6667	0.4193	0.126*	
H8C	0.6124	0.6442	0.4312	0.126*	
C9	0.5430 (6)	0.3069 (9)	0.4576 (7)	0.1194 (19)	
H9A	0.5635	0.3436	0.5218	0.143*	
H9B	0.4823	0.3241	0.4349	0.143*	
C10	0.5550 (7)	0.1785 (11)	0.4652 (9)	0.166 (3)	
H10A	0.5250	0.1455	0.5095	0.250*	
H10B	0.6148	0.1607	0.4891	0.250*	
H10C	0.5335	0.1412	0.4022	0.250*	
C11	0.5383 (5)	0.3602 (8)	0.2873 (7)	0.102 (3)	
H11A	0.5274	0.2740	0.2685	0.123*	
H11B	0.5730	0.3952	0.2478	0.123*	
C12	0.4542 (6)	0.4289 (10)	0.2661 (11)	0.170 (5)	
H12A	0.4257	0.4217	0.1980	0.255*	
H12B	0.4645	0.5149	0.2827	0.255*	
H12C	0.4190	0.3939	0.3041	0.255*	
C13	0.6850 (5)	0.5640 (8)	0.1353 (6)	0.106 (3)	
H13A	0.6629	0.6002	0.0720	0.159*	
H13B	0.6484	0.5852	0.1762	0.159*	
H13C	0.6871	0.4755	0.1293	0.159*	
C14	0.7685 (6)	0.7484 (6)	0.1864 (5)	0.099 (3)	
H14A	0.7449	0.7828	0.1227	0.148*	
H14B	0.8251	0.7804	0.2126	0.148*	
H14C	0.7333	0.7708	0.2285	0.148*	
C15	0.8280 (5)	0.5752 (7)	0.1178 (5)	0.079 (2)	
H15A	0.8251	0.6371	0.0676	0.095*	
H15B	0.8083	0.4970	0.0861	0.095*	
C16	0.9170 (5)	0.5619 (6)	0.1763 (5)	0.079 (2)	
H16A	0.9380	0.6421	0.2032	0.095*	
H16B	0.9519	0.5351	0.1342	0.095*	
C17	1.0132 (5)	0.4808 (8)	0.3220 (6)	0.097 (2)	
H17A	1.0536	0.4534	0.2878	0.145*	
H17B	1.0175	0.4293	0.3782	0.145*	
H17C	1.0251	0.5653	0.3423	0.145*	
C18	0.9109 (5)	0.3440 (6)	0.2185 (5)	0.087 (2)	
H18A	0.9555	0.3209	0.1891	0.131*	
H18B	0.8569	0.3401	0.1707	0.131*	
H18C	0.9106	0.2882	0.2710	0.131*	

### Atomic displacement parameters (Å^2^)

**Table:**

	*U*^11^	*U*^22^	*U*^33^	*U*^12^	*U*^13^	*U*^23^
Fe1	0.0509 (5)	0.0347 (4)	0.0478 (4)	−0.0028 (3)	0.0164 (3)	−0.0015 (3)
Si1	0.0617 (11)	0.0552 (10)	0.0614 (10)	0.0002 (8)	0.0253 (9)	−0.0016 (7)
Cl1	0.0842 (12)	0.0554 (9)	0.0855 (11)	−0.0140 (8)	0.0114 (10)	−0.0227 (8)
N1	0.052 (3)	0.035 (2)	0.060 (3)	−0.001 (2)	0.019 (2)	0.000 (2)
N2	0.086 (4)	0.094 (4)	0.127 (4)	−0.019 (3)	0.047 (3)	0.011 (3)
N3	0.068 (3)	0.060 (3)	0.057 (3)	0.007 (3)	0.013 (3)	0.010 (2)
N4	0.063 (3)	0.060 (3)	0.063 (3)	0.006 (2)	0.030 (3)	0.004 (2)
C1	0.049 (3)	0.042 (3)	0.043 (3)	−0.002 (2)	0.018 (3)	−0.002 (2)
C2	0.066 (4)	0.047 (3)	0.052 (3)	−0.005 (3)	0.019 (3)	−0.005 (2)
C3	0.065 (4)	0.062 (4)	0.055 (3)	0.011 (3)	0.019 (3)	0.012 (3)
C4	0.087 (5)	0.043 (3)	0.076 (4)	0.009 (3)	0.037 (4)	0.007 (3)
C5	0.080 (5)	0.041 (3)	0.103 (5)	−0.010 (3)	0.032 (4)	−0.018 (3)
C6	0.057 (4)	0.054 (3)	0.073 (4)	−0.005 (3)	0.012 (3)	−0.016 (3)
C7	0.108 (6)	0.116 (6)	0.065 (4)	0.014 (5)	0.032 (4)	−0.007 (4)
C8	0.084 (5)	0.062 (4)	0.119 (6)	0.013 (4)	0.050 (5)	−0.002 (4)
C9	0.107 (4)	0.114 (4)	0.143 (5)	−0.024 (4)	0.043 (4)	0.020 (4)
C10	0.151 (6)	0.146 (6)	0.186 (6)	−0.016 (6)	0.015 (6)	0.038 (6)
C11	0.062 (5)	0.099 (6)	0.135 (7)	−0.013 (4)	0.006 (5)	0.007 (5)
C12	0.087 (7)	0.137 (9)	0.260 (15)	0.010 (7)	0.001 (9)	0.013 (9)
C13	0.087 (6)	0.140 (7)	0.075 (5)	−0.002 (5)	−0.006 (5)	0.015 (5)
C14	0.131 (7)	0.059 (4)	0.102 (6)	0.027 (4)	0.022 (5)	0.034 (4)
C15	0.098 (6)	0.083 (5)	0.063 (4)	0.013 (4)	0.034 (4)	0.021 (3)
C16	0.092 (6)	0.076 (4)	0.085 (5)	0.002 (4)	0.049 (4)	0.016 (4)
C17	0.060 (5)	0.128 (7)	0.102 (6)	0.022 (4)	0.022 (4)	0.007 (5)
C18	0.128 (7)	0.072 (4)	0.082 (5)	0.022 (4)	0.064 (5)	−0.001 (4)

### Geometric parameters (Å, º)

**Table:**

Fe1—N1	1.943 (4)	C8—H8B	0.9600
Fe1—N4	2.191 (5)	C8—H8C	0.9600
Fe1—N3	2.215 (4)	C9—C10	1.399 (11)
Fe1—Cl1	2.2798 (16)	C9—H9A	0.9700
Si1—N1	1.713 (5)	C9—H9B	0.9700
Si1—N2	1.726 (7)	C10—H10A	0.9600
Si1—C8	1.872 (7)	C10—H10B	0.9600
Si1—C7	1.876 (7)	C10—H10C	0.9600
N1—C1	1.414 (6)	C11—C12	1.513 (11)
N2—C9	1.464 (10)	C11—H11A	0.9700
N2—C11	1.481 (9)	C11—H11B	0.9700
N3—C15	1.470 (8)	C12—H12A	0.9600
N3—C14	1.474 (8)	C12—H12B	0.9600
N3—C13	1.483 (9)	C12—H12C	0.9600
N4—C16	1.477 (8)	C13—H13A	0.9600
N4—C18	1.479 (8)	C13—H13B	0.9600
N4—C17	1.481 (9)	C13—H13C	0.9600
C1—C2	1.386 (7)	C14—H14A	0.9600
C1—C6	1.387 (7)	C14—H14B	0.9600
C2—C3	1.392 (8)	C14—H14C	0.9600
C2—H2A	0.9300	C15—C16	1.473 (10)
C3—C4	1.365 (8)	C15—H15A	0.9700
C3—H3A	0.9300	C15—H15B	0.9700
C4—C5	1.375 (9)	C16—H16A	0.9700
C4—H4A	0.9300	C16—H16B	0.9700
C5—C6	1.379 (8)	C17—H17A	0.9600
C5—H5A	0.9300	C17—H17B	0.9600
C6—H6A	0.9300	C17—H17C	0.9600
C7—H7A	0.9600	C18—H18A	0.9600
C7—H7B	0.9600	C18—H18B	0.9600
C7—H7C	0.9600	C18—H18C	0.9600
C8—H8A	0.9600		
			
N1—Fe1—N4	119.58 (18)	C10—C9—N2	113.4 (9)
N1—Fe1—N3	114.07 (19)	C10—C9—H9A	108.9
N4—Fe1—N3	81.55 (19)	N2—C9—H9A	108.9
N1—Fe1—Cl1	124.06 (14)	C10—C9—H9B	108.9
N4—Fe1—Cl1	102.41 (14)	N2—C9—H9B	108.9
N3—Fe1—Cl1	106.74 (14)	H9A—C9—H9B	107.7
N1—Si1—N2	113.9 (3)	C9—C10—H10A	109.5
N1—Si1—C8	107.1 (3)	C9—C10—H10B	109.5
N2—Si1—C8	108.4 (3)	H10A—C10—H10B	109.5
N1—Si1—C7	110.5 (3)	C9—C10—H10C	109.5
N2—Si1—C7	107.7 (4)	H10A—C10—H10C	109.5
C8—Si1—C7	109.1 (4)	H10B—C10—H10C	109.5
C1—N1—Si1	118.1 (3)	N2—C11—C12	113.2 (9)
C1—N1—Fe1	115.5 (3)	N2—C11—H11A	108.9
Si1—N1—Fe1	121.7 (2)	C12—C11—H11A	108.9
C9—N2—C11	113.9 (7)	N2—C11—H11B	108.9
C9—N2—Si1	125.8 (6)	C12—C11—H11B	108.9
C11—N2—Si1	119.9 (5)	H11A—C11—H11B	107.7
C15—N3—C14	110.7 (5)	C11—C12—H12A	109.5
C15—N3—C13	108.8 (6)	C11—C12—H12B	109.5
C14—N3—C13	109.1 (6)	H12A—C12—H12B	109.5
C15—N3—Fe1	107.3 (4)	C11—C12—H12C	109.5
C14—N3—Fe1	109.6 (4)	H12A—C12—H12C	109.5
C13—N3—Fe1	111.4 (4)	H12B—C12—H12C	109.5
C16—N4—C18	110.7 (5)	N3—C13—H13A	109.5
C16—N4—C17	109.0 (6)	N3—C13—H13B	109.5
C18—N4—C17	109.1 (6)	H13A—C13—H13B	109.5
C16—N4—Fe1	102.6 (4)	N3—C13—H13C	109.5
C18—N4—Fe1	112.0 (4)	H13A—C13—H13C	109.5
C17—N4—Fe1	113.2 (4)	H13B—C13—H13C	109.5
C2—C1—C6	116.7 (5)	N3—C14—H14A	109.5
C2—C1—N1	120.8 (4)	N3—C14—H14B	109.5
C6—C1—N1	122.4 (5)	H14A—C14—H14B	109.5
C1—C2—C3	121.6 (5)	N3—C14—H14C	109.5
C1—C2—H2A	119.2	H14A—C14—H14C	109.5
C3—C2—H2A	119.2	H14B—C14—H14C	109.5
C4—C3—C2	120.3 (6)	N3—C15—C16	110.9 (6)
C4—C3—H3A	119.8	N3—C15—H15A	109.5
C2—C3—H3A	119.8	C16—C15—H15A	109.5
C3—C4—C5	118.9 (6)	N3—C15—H15B	109.5
C3—C4—H4A	120.5	C16—C15—H15B	109.5
C5—C4—H4A	120.5	H15A—C15—H15B	108.0
C4—C5—C6	120.8 (6)	C15—C16—N4	112.5 (6)
C4—C5—H5A	119.6	C15—C16—H16A	109.1
C6—C5—H5A	119.6	N4—C16—H16A	109.1
C5—C6—C1	121.6 (6)	C15—C16—H16B	109.1
C5—C6—H6A	119.2	N4—C16—H16B	109.1
C1—C6—H6A	119.2	H16A—C16—H16B	107.8
Si1—C7—H7A	109.5	N4—C17—H17A	109.5
Si1—C7—H7B	109.5	N4—C17—H17B	109.5
H7A—C7—H7B	109.5	H17A—C17—H17B	109.5
Si1—C7—H7C	109.5	N4—C17—H17C	109.5
H7A—C7—H7C	109.5	H17A—C17—H17C	109.5
H7B—C7—H7C	109.5	H17B—C17—H17C	109.5
Si1—C8—H8A	109.5	N4—C18—H18A	109.5
Si1—C8—H8B	109.5	N4—C18—H18B	109.5
H8A—C8—H8B	109.5	H18A—C18—H18B	109.5
Si1—C8—H8C	109.5	N4—C18—H18C	109.5
H8A—C8—H8C	109.5	H18A—C18—H18C	109.5
H8B—C8—H8C	109.5	H18B—C18—H18C	109.5
